# Cardiotoxicity of Anticancer Therapeutics

**DOI:** 10.3389/fcvm.2018.00009

**Published:** 2018-02-07

**Authors:** Jerry Dong, Hong Chen

**Affiliations:** ^1^Cardiovascular Biology Program, Oklahoma Medical Research Foundation, Oklahoma City, OK, United States; ^2^Case Western Reserve University, Cleveland, OH, United States; ^3^Department of Surgery, Vascular Biology Program, Boston Children’s Hospital, Harvard Medical School, Boston, MA, United States

**Keywords:** cardiotoxicity, anticancer therapies, signaling pathway inhibitors, chemotherapy, immunotherapy, cardiotoxicity early detection and prevention

## Abstract

As cancer therapeutics continues to improve and progress, the adverse side effects associated with anticancer treatments have also attracted more attention and have become extensively explored. Consequently, the importance of posttreatment follow-ups is becoming increasingly relevant to the discussion. Contemporary treatment methods, such as tyrosine kinase inhibitors, anthracycline chemotherapy, and immunotherapy regimens are effective in treating different modalities of cancers; however, these reagents act through interference with DNA replication or prevent DNA repair, causing endothelial dysfunction, generating reactive oxygen species, or eliciting non-specific immune responses. Therefore, cardiotoxic effects, such as hypertension, heart failure, and left ventricular dysfunction, arise posttreatment. Rising awareness of cardiovascular complications has led to meticulous attention for the evolution of treatment strategies and carefully monitoring between enhanced treatment effectiveness and minimization of adverse toxicity to the cardiovasculature, in which psychological assessments, early detection methods such as biomarkers, magnetic resonance imaging, and various drugs to reverse the damage from cardiotoxic events are more prevalent and their emphasis has increased tremendously. Fully understanding the mechanisms by which the risk factors action for various patients undergoing cancer treatment is also becoming more prevalent in preventing cardiotoxicity down the line.

## Introduction

In the past few decades, anticancer treatment has achieved remarkable progress in improving the quality of life and survival rates of cancer patients. The exploration and application of novel cancer therapeutics have increased tremendously, paralleled with the growth and abundance of literature surrounding the mechanisms underlying cancer metastasis. Novel drug therapies focusing on targeting signaling pathways pertaining to angiogenesis to prevent cellular proliferation *via* kinase inhibitors have especially been promising. Other methods, including anthracycline chemotherapy, have dramatically improved the outcomes of cancer treatment over the last 10 years (1). However, accompanying with the significant improvements toward cancer treatment, cardiotoxicity-related adverse effects caused by these anticancer therapies, specifically on deleterious cardiovascular effects, such as hypertension, heart failure, QT interval prolongation, and left ventricular dysfunction (LVD) (2–5), as well as heart failure with preserved preserved left ventricular ejection fraction (HFpEF), are increasingly reported (6). The development of cardiotoxicity has also been associated with patient age, existing health conditions (at risk of cardiac dysfunction), treatment dosage, and other risk factors (7). Thus, carefully monitoring the development, early detection and prevention of cardiotoxicity, as well as understanding of the interaction between cancer and the cardiovascular system, thereby promoting the development of safer cancer therapeutics, without or with minimized cardiotoxicity, are urgently needed (5).

In this review, we discuss contemporary methods of cancer therapy and the related signaling pathways, which are promoting heart dysfunction and are affected through inhibitory drug treatments that are often compounded with chemotherapy and radiotherapy. We also discuss the importance of the novel therapeutic detection approaches for cardiotoxicity, namely, early detection *via* biomarkers and cardiovascular imaging, including, but not limited to, magnetic resonance imaging. These alternative treatment routes may provide more insight into the efficacy of cancer treatment strategies and cancer diagnostic tools, which highlight the importance of early detection to avoid later onset of adverse cardiotoxic effects.

## Cardiotoxicity by Anticancer Treatment

The National Cancer Institute defines cardiotoxicity as “toxicity that affects the heart.” Cardiotoxicity may be acute, which occurs during or soon after treatment and is transient, or chronic, and can be categorized into type I (early onset) and type II (late onset) (5). Type I is irreversible cardiac cell injury and usually caused by anthracyclines and chemotherapeutics; type II is typically caused by novel biological-targeted antibodies (8). Chemotherapy and metabolic pathway inhibition has been shown to create adverse side effects, predominantly focusing on myocardial damage and the risks associated with heart failure posttreatment, although the newly emerged targeted drugs such as tyrosine kinase inhibitors and antibodies induce toxicities different from chemotherapy. Often, cardiotoxicity is commonly associated with LVD and other symptoms of systemic heart failure. Furthermore, LVD condition has several facets, which can be related to myocardial toxicity but also to other cardiovascular toxicities, namely, QTc prolongation, arrhythmia, myocardial ischemia induced through atherosclerosis, and pulmonary hypertension. Especially, HFpEF (also called diastolic heart failure) occurs when the lower left ventricle is unable to properly fill with blood during the diastolic phase, and increasingly arises in patients undergoing chemotherapy, and may become predominant HF. Because the pathophysiology underlying HFpEF is heterogeneous, has different phenotypes, and is poorly understood, the etiological definition of HFpEF is variable. Thus, accurate diagnosis is challenging, and currently there is no effective therapy for HFpEF (6, 9–11). However, recently identified novel biomarkers, such as protein biomarker of cardiac stress (ST2), matrix metalloproteinase-2, and growth differentiation factor-15, for the risk stratification of HFpEF may be used for development of significant therapeutic targets for the treatment of HFpEF (12). Furthermore, recent advancements in imaging techniques and exploration into biomarkers have raised the important issue of the multiple comorbidities of cardiotoxicity, many of which are not agglomerated through drug therapies.

Currently, there is no consensus definition of cardiotoxicity. The Cardiac Review and Evaluation Committee of trastuzumab-associated cardiotoxicity defines cardiotoxicity as symptoms of heart failure, decline of left ventricular ejection fraction (LVEF), symptomatic fall in LVEF ≥5 to <55% or an asymptomatic reduction of LVEF ≥10 to <55% (13). The American Society of Echocardiography and European Association of Cardiovascular Imaging define cardiotoxicity as global longitudinal strain (GLS) with a 10–15% early reduction (http://asecho.org/wordpress/wp-content/uploads/2015/12/MLM-Revised-Strain-Code-11-12-15.pdf). The Food and Drug Administration defines LVEF drop <40–45% or is 40–49% with a ≥10% absolute decrease below baseline with anti-HER2 targeted therapy as being necessary to be monitored (14).

The Common Terminology Criteria for Adverse Events (CTCAE) is a descriptive terminology that is used for adverse event reporting based on a grading scale ranging from 1 to 5, with 1 being abnormal elevations in biomarker expression or imaging abnormalities, to 5, death due to cardiotoxicity (CTCAE, version 4.0 National Cancer Institute, June 14, 2010). However, the current reporting scale may be confusing and depends highly on symptomology, which may not represent a clear image of LVD abnormalities, for example, with patients who are asymptomatic. Finally, attributing signs and symptoms of cardiotoxicity prove to be difficult, as many symptoms are may not be induced or attributable to the drug therapy itself. This is especially challenging in older patients, whom often have common comorbidities.

Although the current scale may be deficient in some ways, it is important to address the limitations of potential definitions. Research of this magnitude requires consistency in defining the issue to share a common language and reinforce validity in the research.

## Contemporary Therapeutics for Neoplasia

Currently, immunotherapies use the immune system to enhance their antitumor immunity and further immune responses by employing immune checkpoint inhibitors, chimeric antigen receptor (CAR) T cell therapy, and adaptive cell transfer (ACT) (using the patient’s own T-cells, engineered to specifically target cancer cells) have been promising in certain cancer treatments (15). Other targeted therapies, specifically focusing on signaling pathway inhibition to prevent certain cell processes from occurring, namely, angiogenesis, also improve clinical outcomes. Angiogenesis, a normal process in which blood vessels are created through currently existing vessels, is a vital process in wound healing, growth, and development. However, growing tumors hijack this process to feed and proliferate tumor cells, thereby creating malignant tumor vessels within the body, making angiogenesis a key factor for tumor growth and survival.

One of the most prominently used and evaluated strategies is the inhibition of the vascular endothelial growth factor (VEGF) signaling cascade. VEGF plays a critical role in angiogenesis through binding VEGF receptors and activating the VEGF signaling pathway. Inhibition of angiogenesis, thereby, prevents growing tumors from hijacking the body’s natural process (16). Another popular target for cancer therapies is the human epidermal growth factor receptor 2 (HER2). The HER2 protein, also called ERBB2, is commonly overexpressed in patients with breast cancer, which accounts for approximately 15–30% cases in breast cancer (17, 18). Normally, HER2 helps in growth, proliferation, and repairing of abnormal cells within the body. However, similar to the VEGF signaling pathway, tumorigenesis takes control of the cellular process and promotes the proliferation of cancer cells (19, 20).

Trastuzumab (Herceptin), a monoclonal antibody, specifically targets HER2. Treatment regimens with Trastuzumab, coupled with chemotherapy, have shown remarkable outcomes in patients with breast cancer after 1 year (21). Anthracycline antibodies, including doxorubicin (DOX), have also been a consistent and prominent form of chemotherapy schemes for nearly half a century. By limiting proliferation of cancer cells *via* preventative interference with its DNA or RNA structure, DOX is able to halt tumorigenesis and ultimately stop cancer cell proliferation and division (8, 22).

## Targeting the VEGF Signaling Pathway

Vascular endothelial growth factor and its corresponding receptors, VEGFR, are one of the most important tyrosine kinase pathways. VEGF includes seven members in its family—VEGF-A, VEGF-B, VEGF-C, VEGF-D, VEGF-E, VEGF-F, and PIGF. VEGF-A is the most representative component. VEGF-A mRNA is expressed in various tissues in the body, such as the lung, kidney, and heart (16). Consequently, the VEGF signaling pathway plays a central role in other signaling pathways which also affect the vasculature, and any alterations to this pathway have been shown to exhibit deleterious effects (23).

Mechanistically, VEGF binds to three receptor sites—VEGFR-1 (Flt-1), VEGFR-2 (Flk-1), and VEGFR-3 (24). Binding of VEGF to VEGFR-2 initiates the tyrosine signaling cascade, which then facilitates cell migration, proliferation, growth, and vasodilation, all of which are important and contribute to the development of angiogenesis (25). Historically, the VEGF pathway can be targeted using several different methods, including the molecule itself (monoclonal antibodies), receptors (recombinants), or downstream signaling pathways and inhibiting downstream expression (tyrosine kinase inhibition) (4).

Current antiangiogenesis therapies, including the inhibition of VEGF, have shown to have adverse effects on the cardiovascular system (2, 7, 16). VEGFR-1 and VEGFR-2 are both expressed naturally in endothelial cells (EC) (26). Due to the nature of inhibitory drug therapies, affecting the expression and interaction between VEGF and its receptor sites affects the entire circulatory system and primarily induces proliferation of EC and promoting vascular integrity. Anti-VEGF therapies, such as the tyrosine kinase inhibitor, imatinib, dasatinib, nilotinib, bosutinib, sunitinib, sorafenib, axitinib, and ponatinib, exacerbate LVD, as well as incidences of hypertension, ischemic events, rapid acceleration of atherosclerosis, and other vascular toxic events (27). Inhibition of the VEGF pathway, therefore, can lead to endothelial dysfunction, arising from the disruption of normal endothelial homeostasis mediated through nitric oxide (23).

Meta-analyses with sunitinib have shown increased relative risk for cardiovascular complications. In a meta-analysis of 9,387 patients, the risk of myocardial ischemia caused by sunitinib was 3.03-fold higher compared with placebo (28). Sunitinib has also been speculated to induce LVD risk in over 20% of patients (28). Ponatinib has been associated with significant cardiovascular toxicity, with an indication of 10% cardiovascular, 7% cerebrovascular, and 7% peripheral adverse events by 28 months after treatment. Hypertension was reported in 26% of patients treated with this drug (28).

## Targeting the HER2 Signaling Pathway

Trastuzumab is a humanized monoclonal antibody that directly inhibits the HER2 signaling pathway. It is used as first-line therapy with chemotherapy and has been shown to be effective in 25–30% of breast cancer cases (5, 29). However, anti-HER2 therapy was reported to increase the risk for asymptomatic decreased left ventricular ejection fraction, leading to complications such as systemic heart failure. Interestingly, the cardiotoxic effects are shown early (usually within weeks of initial treatment) and are independent of dosing regimens. The cardiotoxic effects are also type II, or reversible, and often not severe in terms of cell death or damage (3).

Mechanistically, Trastuzumab competes for binding sites on the extracellular domain of HER2 and inhibits the activation of the tyrosine kinase signaling pathway (30, 31). It has also been speculated that Trastuzumab also induces cell death *via* antibody treatment, although the data supporting the claim are not yet clear (32). Regardless, HER2 inhibition prevents cell repair and significantly limits proliferation. However, it is speculated that because Trastuzumab’s effect is established through targeting the HER2 signaling pathway, which regulates cell differentiation, survival, and repair in healthy tissues, the body’s myocytes cannot depend on the repair mechanism either, and that leads to cardiotoxicity when in the presence of anthracyclines (33). Supporting data also come from a study of 179 breast cancer patients, in which 44% of patients developed a cardiac event (e.g., he art failure or decreased ejection fraction) and a tenth developed a second event (34, 35).

## Anthracyclines

Anthracyclines, a class of chemotherapy drugs, are traditional cancer therapies that have been effective in treating many forms of cancer for the last half century. One of the most prominently used and readily identifiable is the anthracycline DOX. DOX-induced cardiotoxicity is classified as type I, or irreversible. It directly contrasts with Trastuzumab and HER2 inhibition by the fact that cardiotoxicity from DOX is dose dependent (usually doses ≥450 mg/m^2^) (36). Moreover, the risk for future complications significantly increases with cumulative doses (37, 38). Although it was originally speculated that the risks for immediate, on-treatment events decreased with lower doses, it is becoming more apparent that late onset complications can be verified regardless of dose. Therefore, there is no dosage regimen of DOX that is completely safe. The immediate cardiotoxic effects can range from a few weeks to years after treatment, even after the treatment has been discontinued. Incidences and reporting of LVD complications range from 1 to 20%, although the rate may be much higher (39). Some studies estimate that over half of the patients exposed to anthracyclines will develop some forms of LVD within 6 years (40). This claim is further supported by recent studies reporting the incidence of cardiotoxicity in 17.9 and 6.3% of patients for subclinical and overt cardiotoxicity, respectively, in 9 years (41). Other studies have also shown LVEF in 98% of patients in a cohort of 9% (*n* = 226) overall incidence for cardiotoxicity within 1 year after chemotherapy (42).

Chemically, DOX has both hydrophilic and hydrophobic regions, allowing it to bind to both plasma proteins and cell membranes. DOX also has both acidic and base functions, its characteristics of being reoxidized results in the production of reactive oxygen species (ROS) and gives DOX an antineoplastic and antibiotic capabilities (8, 43). DOX has several different channels in which it affects the overall homeostasis in the body. One of the most important is its interaction with topoisomerase II. Topoisomerases are isomerase enzymes that participate in the overwinding or underwinding of DNA, allowing DNA to replicate by binding to the double strand DNA and overcome the tangles caused by the double helix. Topoisomerase II has two nuclear localized isoforms: topoisomerase IIα and IIβ. DOX inhibits topoisomerase interaction with DNA by directly binding to both IIα and IIβ, forming a DNA cleavage complex that increases double strand breakage (8, 22). Although DOX prevents cancer cells from replicating, it also serves as the primary cytotoxic reagent to induce cell apoptosis. DOX affects calcium homeostasis as well. It directly interferes with calcium storage capacity of the mitochondria by activating the selective CsA-sensitive calcium channel, causing calcium overload (8, 44) and leads to mitochondrial dysfunction, as well as apoptosis. The decreased calcium levels from the mitochondria have been shown to be irreversible. DOX, therefore, is highly versatile in terms of its uses, as well as its impact on the vasculature, and the cardiovascular system as a whole.

## Cancer Immunotherapy

Cancer immunotherapy is a newly emerging treatment method which bases itself on the deeper understanding of the mechanism of antitumor immune responses, discoveries of novel anticancer molecules (peptides and vaccines), and development of innovative technologies of gene transfer (45). Current popular cancer immunotherapy employing inhibitory effects to immune checkpoint receptors has proven to be very effective in several malignancies and has shown very promising clinical outcomes in various types of cancers in the past 10 years. This revolutionized strategy has brought anticancer treatment into a new era (8, 45). Anti-cytotoxic T lymphocyte-associated antigen 4 (CTLA-4) and anti-programmed cell death protein-1 (PD-1) have especially been beneficial to ailments, such as melanoma (46). Normally, cancer cells can utilize these receptors to avoid destruction *via* T cells in the immune system by binding to CLTA-4 and reducing naïve T cell activation or by expressing the cell death protein ligand-1 (PD-L1), which then binds to PD-1 and mediates T cell downregulation and apoptosis. Inhibitors, such as ipilimumab (anti-CTLA-4 monoclonal antibody) and pembrolizumab and nivolumab (anti-PD-1 monoclonal antibodies), bind to and block the inhibitory effects of the receptor sites, thus enhancing the cytotoxic immune response to the cancer cells (45).

Adoptive T cell transfer (ACT) is another novel and a promising method for a wide spectrum of solid cancer treatment. ACT uses a patient’s T cells to specifically target tumor cells. Mechanistically, tumor infiltrating lymphocytes (TILs) are collected and genetically engineered into a patient’s microenvironment along with systemic interleukin-2 (IL-2) to stimulate their survival and expansion (45). Successful implementation of ACT as a treatment modality for patients with metastatic melanoma has established the basis for multiple modifications and improvements of this strategy for targeting many different cancers (47). It has been reported that in 50% of patients with metastatic melanoma exhibit objective tumor regression by ACT using autologous TILs (47). As an innate cytokine in the immune system with essential and wide spectrum of immunological functions, IL-2 has long been used for cancer treatment alone and has shown great tumor regression (48, 49). IL-2 directly regulates T cell differentiation into different T cells in response to antigens to enhance the defense of the immune system (48, 49). A recent report of a clinical study with patients treated with lymphodepleting conditioning chemotherapy followed by infusion of autologous TILs and high-dose IL-2 showed regression of metastatic uveal melanoma in 7 out of 20 patients, which had been believed to be resistant to immunotherapy previously (50). Similarly, chimeric antigen receptor (CAR)-T therapy has had valuable benefits to certain deficiencies, such as B cell acute lymphoblastic leukemia (B-ALL). Mechanistically, CAR-T functions very similar to ACT, wherein native T-cells are engineered to express a chimeric antigen receptor on the cell membrane. The receptor is coupled with an external binding domain to specifically recognize and bind to tumor antigens. CAR-T also has an internal activation domain that then activates the T-cell once CAR-T binds to its target. In patients with B-ALL, CAR-T therapy is effective in 70–90% of cases (51).

However, the therapeutic effects are also counterbalanced with similar cardiotoxic effects (52–54). This is especially the case with immunotherapies, since activated T cell responses may be non-specific to cancer cells, thereby targeting normal tissue as well, leading to frequent immune-related adverse events (IRAEs) such as colitis, endocrinopathies, hepatitis, and pneumonitis. Other events, such as cardiomyopathy, myocardial fibrosis, myocarditis, and acute heart failure, were also reported in single anti-CTLA-4 or anti-PD-1 treatment (54), and lethal myocarditis accompanying with myositis was seen in combinatorial treatment (55), although such cases are rare. Patients using the monoclonal antibody ipilimumab have an IRAE frequency rate of 64–80% (54, 56, 57). Patients treated with pembrolizumab have an IRAE frequency rate of up to 79% (54, 58). Combination therapies using both ipilimumab and nivolumab have been shown to have tremendous therapeutic efficacy and response rates in patients with melanoma from 19% using ipilimumab alone to 58% with the combination (46, 52). However, the frequency rate of IRAEs also increased parallel to the combination therapy, reaching as high as 96% in patients using both ipilimumab and nivolumab (59). CAR-T therapy and ACT both have associated risks with cardiotoxicity. CAR-T therapy, specifically, has links with cytokine release syndrome (CRS), an inflammatory response that is correlated with the activation of CAR-T cells. Manifestations of CRS include arrhythmias, decreased LVEF, and QT prolongation (60).

## Prevention of Cardiotoxicity and Future Directions

Reversing certain effects of cancer drug therapies may not always be possible. However, current research into managing and monitoring cardiotoxicity and the various side effects arising from both modern and traditional therapeutics have been very promising. Dexrazoxane, a most promising cardioprotective agent, has been shown to be effective in reducing both acute and chronic cardiotoxicity induced by anthracycline therapy (8, 61). Dexrazoxane has been shown to interfere with iron-dependent redox reactions, thereby reducing ROS originating from DOX (62). Dexrazoxane also directly inhibits topoisomerase IIβ, thereby preventing anthracycline binding and DNA double strand breaks (63). However, the ability of Dexrazoxane in reducing anthracycline-induced cardiotoxicity has been limited in its clinical use because of adverse side effects (5). A recent clinical study indicated that Dexrazoxane, in addition to DOX, resulted in higher rates of bone marrow suppression, more febrile neutropenia events and dose reductions (64). Seeking novel protection of cardiotoxicity drugs is thereby more essential and in urgent demand.

Treatments modalities employed either before, after, or before and after cancer therapeutics have proven effective when dealing with certain cardiotoxic effect risk factors, such as heart failure. The use of beta blockers (BBs), angiotensin-converting enzyme (ACE) inhibitor, angiotensin inhibitors, and mineralocorticoid receptor antagonists have all shown promising results in preventing cardiac damage and is an active ongoing investigation area (7). BBs have antioxidant properties and were utilized for chemotherapy-induced LVEF and other systolic LV dysfunction. One of the new generation BBs and a most commonly used agent, carvedilol, showed strong antioxidant characteristics and greater protective effect on anthracycline-induced cardiomyopathy (6, 8). A recent report demonstrated that favorable effects of ACE inhibitors and BBs on preventing cardiotoxicity and improving survival of breast cancer patients treated with trastuzumab and/or anthracyclines (65). Moreover, beta-adrenergic blockade with nebivolol, metoprolol, lisinopril, etc., has also been proven to be effective for cardiomyopathy (66, 67). However, reports on some BBs demonstrating beneficial cardioprotective effects in patients with LVEF remains controversial (68). Novel modalities, such as lcz696 (valsartan/sacubitril) (69, 70) and ivabradine can also be used to treat heart failure and subsequent impaired myocardial systolic dysfunction (6, 71). Furthermore, the cardioprotective role of ranolazine (inhibition of late INa elevation) and phosphodiesterase-5 inhibitors has been reported (66). In addition, nutrition supplements and exercise have shown positive effects on cardiomyopathy (66). However, significant supportive clinical data are still needed to prove the protective function of these novel agents in patients affected by cardiotoxic effects (7).

The role of biomarkers has also been important in early detection. Elevated or abnormal expression levels of several biomarkers can be used as indicators for screening and assess the risk factors for future cardiotoxicity complications. Interleukin-6 (IL-6), a cytokine produced by adipose tissues, increases blood pressure and induces inflammation. Overexpression of IL-6 inhibits cell apoptosis, stimulates angiogenesis, and plays a role in drug resistance (72). There have also been promising data exploring biomarkers that pertain to type I and type II cardiac events. Increases in troponin-I, brain-type natriuretic peptide (BNP), and N-terminal-pro-BNP have all been linked to drops in LVEF (5, 73). Plasma myeloperoxidase also predicts a decrease in cardiac function (7). MicroRNA has emerged as a potential marker for early onset of heart failure. miR-1, miR-133b, and miR-146a were all upregulated corresponding to DOX chemotherapy (74).

Early detection is vital when considering the deleterious effects that drug therapeutics may have. One facet in which early detection has been outlined is through the risk factors for both cardiovascular diseases and cancer. Shared risk factors, such as obesity, carcinogenic agent usage (e.g., tobacco, smoking, and alcohol), previous history of diabetes, hypertension, non-modifiable risk factors (e.g., age and sex), and physical activity, should all be taken into consideration, especially when pertaining to drug therapeutics, thereby minimalizing the risk of future cardiotoxicity events (75).

Current exploration into psychological stress as an additional risk factor is promising (76). The mechanism in which psychological distress plays in cardiovascular dysfunction is twofold, focusing on behavioral and pathophysiological. In terms of behavioral mechanisms, it is best understood that personality types, temperament, anxiety, and depression can influence and are related to other risk factors, such as unhealthy lifestyles, lack of exercise, smoking, and alcohol consumption (77). In terms of pathophysiological mechanisms, being under psychological distress may form complications such as platelet dysfunction, autonomic nervous system dysregulation, hypothalamic–pituitary–adrenal axis (HPA-axis) dysregulation, cellular aging, and inflammatory activation. Platelet activation has shown increases in patients with depression (78). The HPA-axis has a known role and association in cardiovascular dysfunction *via* cortisol regulation (79). Patients with depression, anxiety, and fatigue often show elevated cortisol levels (80). Patients with depression and anxiety also have shorter telomere length, a biomarker for cellular aging and also an associated risk factor for cardiovascular dysfunction (81).

Takotsubo cardiomyopathy (TC), known as stress cardiomyopathy or broken heart syndrome, may be caused by emotional stress and long-term anxiety (82). TC shows similar images as that of acute coronary syndrome and is characterized by dynamic electrocardiographic changes along with transient, severe, and reversible LVD, but unclear pathophysiological mechanisms (82, 83). It is more common in elder women and was taken into account only in recent years in cancer patients treated with anticancer drugs suggesting that chemotherapy can induce TC (83–85).

Detection and early screening of cardiotoxicity *via* imaging techniques have become more prevalent. Two-dimensional echocardiography (2DE) has been the standard for quite some time. However, assessment of LVEF and GLS *via* three-dimensional echocardiography (3DE) and speckle-tracking echocardiography, respectively, has been shown to be a valuable asset in early detection and is able to overcome many challenges that affect traditional 2DE methods (7, 86). Moreover, GLS seems to predate LVEF decreases, and impairment of GLS in HEpEF has been reported (87). Thus, GLS seems a better indicator of myocardial dysfunction prior heart failure progression. Cardiac magnetic resonance imaging has emerged in recent years and is used as the gold standard parameter. It is accurate, reproductive, and reliable and has higher sensitivity than 2DE and 3DE in detecting early changes in global and regional cardiac function, and its high contrast-to-noise ratio provides excellent structural characteristics (88, 89).

## Conclusion

Cardiotoxicity is one of the most deleterious effects arising from cancer therapeutics and a major barrier to survivorship. However, today’s cancer patients should not be tomorrow’s cardiovascular disease patients. Recent research has shed the light of optimism with an emphasis on early prevention. Continued research and discussion will further advance our literature pertaining to cardiotoxicity, opening up additional avenues for safer treatment strategies. Further investigation into alternative therapeutics, as well as the increasing information and understanding of modern technologies in screening and detecting at-risk patients will be helpful in developing and evaluating different modalities for cancer therapeutics. Moreover, such research will be able to fill in the gaps in understanding cardiotoxicity and explore other avenues of research that are often overlooked.

Understanding the importance of risk factors and associated risks, such as the role of psychological distress, are necessary to advance the care of cancer patients. Minimizing the risk of cardiovascular complications induced through various therapies is vital to treatment and care. This, again, is an emphasis on the importance of early detection and risk assessment when considering drug administration to certain patients.

## Author Contributions

JD and HC wrote the manuscript and finalized the manuscript.

## Conflict of Interest Statement

The authors declare that the research was conducted in the absence of any commercial or financial relationships that could be construed as a potential conflict of interest. The reviewer AF and handling editor declared their shared affiliation.
